# Review of rehabilitation protocols for brachial plexus injury

**DOI:** 10.3389/fneur.2023.1084223

**Published:** 2023-04-17

**Authors:** Haijun Li, Jinxiu Chen, Juehan Wang, Tianfang Zhang, Zuobing Chen

**Affiliations:** Department of Rehabilitation Medicine, The First Affiliated Hospital, Zhejiang University School of Medicine, Hangzhou, China

**Keywords:** brachial plexus injury, rehabilitation, neuropathic pain, neonatal brachial plexus injury, treatment

## Abstract

Brachial plexus injury (BPI) is one of the most serious peripheral nerve injuries, resulting in severe and persistent impairments of the upper limb and disability in adults and children alike. With the relatively mature early diagnosis and surgical technique of brachial plexus injury, the demand for rehabilitation treatment after brachial plexus injury is gradually increasing. Rehabilitation intervention can be beneficial to some extent during all stages of recovery, including the spontaneous recovery period, the postoperative period, and the sequelae period. However, due to the complex composition of the brachial plexus, location of injury, and the different causes, the treatment varies. A clear rehabilitation process has not been developed yet. Rehabilitation therapy that has been widely studied focusing on exercise therapy, sensory training, neuroelectromagnetic stimulation, neurotrophic factors, acupuncture and massage therapy, etc., while interventions like hydrotherapy, phototherapy, and neural stem cell therapy are less studied. In addition, rehabilitation methods in some special condition and group often neglected, such as postoperative edema, pain, and neonates. The purpose of this article is to explore the potential contributions of various methods to brachial plexus injury rehabilitation and to provide a concise overview of the interventions that have been shown to be beneficial. The key contribution of this article is to form relatively clear rehabilitation processes based on different periods and populations, which provides an important reference for the treatment of brachial plexus injuries.

## 1. Introduction

Brachial plexus injury (BPI) is one of the most serious peripheral nerve injuries, with severe physical disabilities and long-term financial and psychological consequences. Epidemiological studies conducted in both developing and developed countries have shown that brachial plexus injuries are very common in productive young men. Closed injury is the most common type of injury, caused primarily by strains, fractures, and compression injuries, medical injuries are also quite common (1). When a newborn sustains a brachial plexus injury, it is referred to as a neonatal brachial plexus injury (NBPI), which is caused primarily by a birth incident. The brachial plexus nerve is a complex neural component that arises from the anterior branches of the cervical (C5-8) and thoracic (T1) segments of the spinal cord and creates an interlocking network that innervates the muscles of the upper extremities and transmits cutaneous sensation. It is challenging to pinpoint the damage precisely because of the intricate structure of brachial plexus nerve. Surgery and neurological rehabilitation employ distinct methods and struggle to provide consistent outcomes. Myelography methods and neurophysiological exams have steadily evolved in recent years. Simultaneously, surgical procedures such as nerve repair and grafting have increasingly matured. There is currently no comprehensive rehabilitation program available for individuals with various degrees of brachial plexus injury. To create a more comprehensive treatment plan, we will incorporate the most relevant and successful rehabilitation therapy approaches for various timeframes. To serve as a reference for the clinical management of brachial plexus injury, we will also briefly discuss the fundamental rehabilitation treatments for each period.

## 2. Brachial plexus injury: Neural localization and clinical symptoms

**Figure 1:**
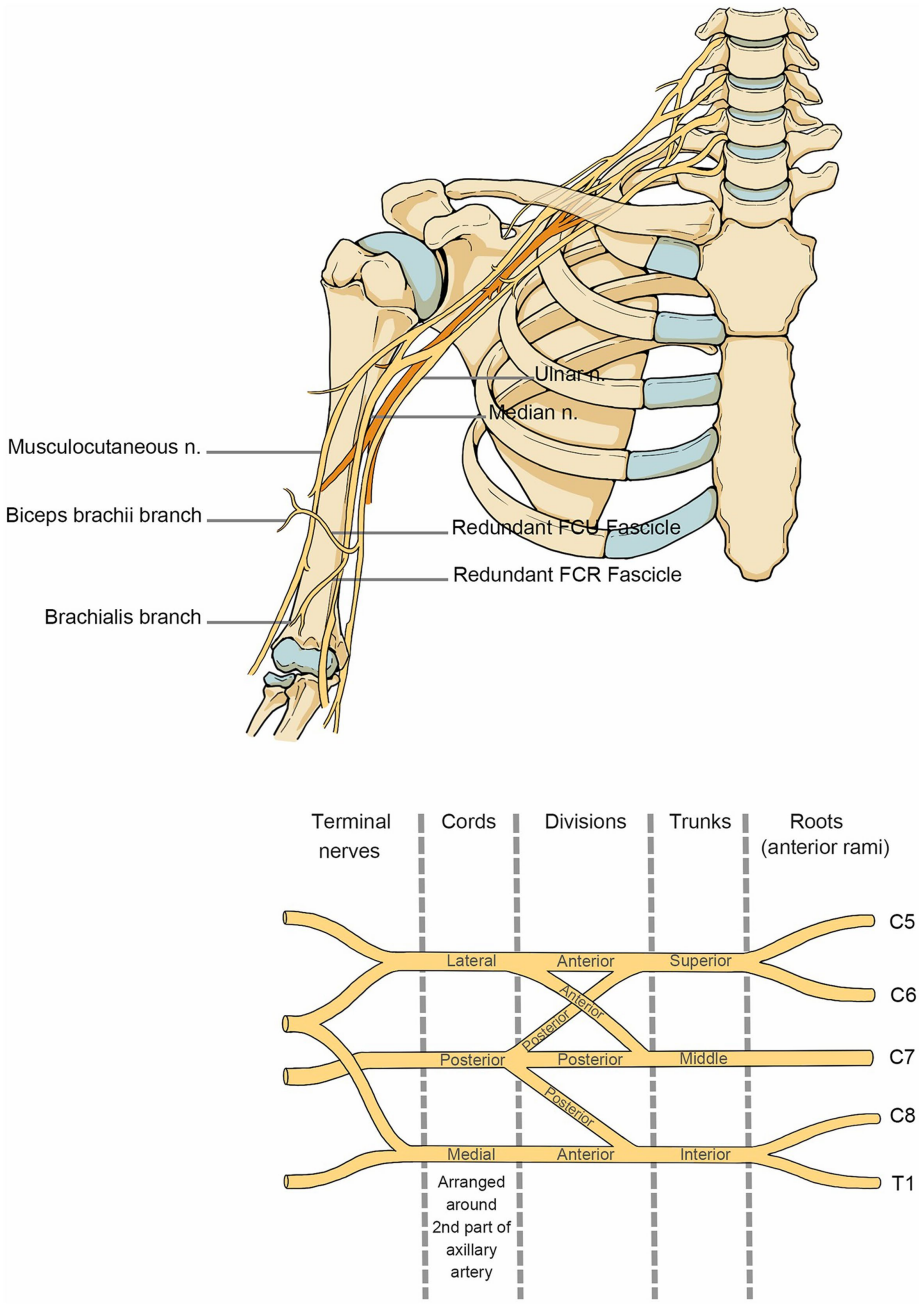
Anatomy of the brachial plexus nerve. The anatomical course of the brachial plexus nerve in the shoulder, neck and upper arm.

Brachial plexus injuries (Figure 1) fall under many categories. However, functional placement is more advised since sensory-motor reconstruction and muscle balance restoration are the main goals of therapy after brachial plexus injury. When the affected nerve is C5 and C6, it is categorized as “superior trunk dissection,” which is characterized by impaired shoulder and elbow flexion, abduction, and external rotation while hand function is preserved, and is also known as Duchenne-Erb syndrome. When the involved nerve is C7, it is categorized as “middle trunk dissection.” When the involved nerve is C8 and T1, it is categorized as “inferior trunk dissection,” which is characterized by the impaired hand and wrist function, also known as Dejerine-Klumpke syndrome. Complete brachial plexus palsy (C5-T1) affects the function of the entire arm, resulting in a fully flaccid arm. When this condition is coupled with an eye injury, it is referred to as Horner’s syndrome.

## 3. Adult brachial plexus injury rehabilitation

The management of BPI depends on the type, location, and severity of the injury. It is usually determined by magnetic resonance imaging and/or computed tomography myelography. Surgeons now agree that the best time for surgery is 3–6 months after injury; traction injuries usually heal spontaneously within 3–6 months, and surgical exploration may cause secondary injury, so surgical intervention is recommended if nerve function has not recovered by the end of the spontaneous recovery period (2). Complete tears, ruptures, and lacerations typically do not recover within this time range; hence, early surgical intervention is advised in place of this. Unsurprisingly, rehabilitation, including neurotrophic therapy, is the best course of action before surgery. Following surgery, rehabilitation treatment can aid in the restoration of neurological function. Early intervention for rehabilitation is necessary since brachial plexus injury, with or without surgery, since BPI will cause some degree of muscle loss in the upper extremity and thus can inevitably culminate in fast muscular atrophy. Promoting nerve regeneration and functional recovery while safeguarding and regaining function in joints and intact muscles is the main objective of brachial plexus injury rehabilitation. Based on this premise, subsequent abnormalities such as muscle atrophy, joint stiffness, and limited range of motion are all minimized to the greatest extent feasible.

### 3.1. Rehabilitation in the spontaneous recovery period

Axonal growth occurs at a pace of approximately 1 mm every day. The basic objectives of the spontaneous recovery period following brachial plexus injury are to ensure adequate muscle nutrition, preserve muscle function, and promote nerve regeneration until the nerve reaches the periphery because it takes a long time for nerves to reinnervate.

#### 3.1.1. Pain management

Pain is present at all stages of brachial plexus injury, including acute neuromuscular injury pain, postoperative pain in the operative area, and chronic neuropathic pain, and it significantly reduces patients’ rehabilitation compliance, mental health, and quality of life. It should be addressed as soon as it is identified. A review by Tom et al. for the functional assessment of brachial plexus injury recommends the use of the Patient Reported Outcome Measure (PROM) preferably for those patients who can report pain autonomously, and the commonly used Visual Analog Scale (VAS) and Numeric Rating Scale (NRS) are also recommended (3). However, there is no efficacy analysis of different pain assessment scales for brachial plexus injury. The various types of pharmaceuticals listed in the overview of pain management after complicated nerve injury (4), such as central analgesics, psychiatric medications, narcotics, and hormones, can be used by both surgical and non-surgical patients. Oral and local injections are just two examples of administration methods. Given that pain is subjective and frequently accompanied by psychological illnesses, psychotherapy, and psychotropic medications may occasionally provide further advantages with lingering effects (5). Yun et al. have demonstrated that continuous interscalene brachial plexus block (CISB) has better effective analgesia, better sleep quality and causes fewer opioid-related complications than single interscalene brachial plexus block (SISB) (6). There have not been many studies comparing the efficacy of various drugs and administration methods. Psychotherapy, along with physiotherapy, exercise therapy, invasive surgery, and others, is one of the non-pharmacological strategies that, when combined with pharmacotherapy, form a comprehensive pain treatment. It is critical to understand that medication is not a substitute for non-drug approaches. Furthermore, while chronic neuropathologic pain is commonly challenging to manage with medication, it can still be used to provide short-term relief in acute nerve injury pain or postoperative pain. Neurostimulation has become increasingly effective for chronic neuropathic pain in recent years, in addition to surgical treatments such as nerve transfer and neuroma excision. Thalamic DBS, for example, has proven to be an effective treatment option for patients suffering from severe and medically incurable pain (7). However, studies on this kind of neural stimulation typically do not include a lot of instances, and there is not a widely accepted standard stimulation paradigm due to the heterogeneity of different stimulation parameters. Future research should be expanded upon, it is advised.

#### 3.1.2. Motor reeducation

Strong external fixation support of the damaged limb is necessary before commencing activities. When shoulder and elbow movements are compromised, shoulder straps and figure-eight bandages can be utilized to restrict the uncontrolled movement of paralyzed joints. When the wrist joint is affected, a splint can be utilized to maintain the wrist in an extended position for 10–20 degrees, assisting in the prevention of joint contracture and easing the patient’s pain (8). The advantages of various types of external fixation have not been studied; thus, the choice may be determined based on the medical profile and tolerance level of the patient.

There is a large amount of literature on motor re-education after brachial plexus injury. Typical therapies include passive range of motion, active-assisted range of motion, active range of motion, and strength, according to a systematic review based on proprioceptive neuromuscular facilitation (PNF) theory (9). This is a progressive procedure that might start with a determination of the strength of the muscles in the upper extremities and the choice of an adequate range-of-motion recovery based on strength grading. The therapeutic concepts indicated in various treatment reports are summarized as follows (10, 11): When the patient has no muscular contraction, a passive range of motion is employed. When the patient maintains sufficient muscular contraction, an active-assisted range of motion is employed. As muscular strength grows, the therapist’s function in joint range of motion is eventually superseded by the patient, and resistance rises.

In comparison to simulative activities or pointless exercise, a systematic study revealed that involvement in purposeful activities for upper extremity motor therapy regularly leads to a greater quantity and quality of muscle building (12). Occupational therapy is frequently utilized after brachial plexus injuries and is based on deliberate and targeted occupational exercises to enhance the patient’s capacity to adapt to daily life and expedite the patient’s return to family and society. A prospective longitudinal study confirmed that postoperative specialist occupational therapy increased motor recovery and upper extremity function in patients following BPI, involving occupational therapy such as folding towels, drinking water, lifting plastic bags, cutting with utensils, etc. (13). The profession of occupational therapy for patients with brachial plexus injuries is not standardized. Nonetheless, it would be beneficial to design more occupational forms that incorporate bending the elbow, bending the shoulder, and extending the wrist, as many doctors expect, which will help the patient regain their capacity to live independently (14). Furthermore, the therapist presses and pulls on the affected limb, or the patient voluntarily touches the object, providing some tactile stimulation that contributes to activity-based sensory recovery. Better outcomes will come from including exposure to different objects at various temperatures during the movement treatment.

#### 3.1.3. Sensory reeducation

Brachial plexus injury can cause sensory deficiencies in the upper extremities, including sensory anomalies and hypoesthesia. Standardized tests can evaluate sensory thresholds and functions in both children and adults including the two-point discrimination test, skin pressure thresholds, vibration perception, and assessments of pain and tactile sensations using needles and cotton (15). Patrick et al. summarized seven rehabilitation techniques for peripheral nerve injuries based on the theory of cortical remodeling. The sensory reeducation component comprises traditional sensory reeducation, activity-based sensory reeducation, mirror visual feedback, cross-modal sensory substitution, and selective deafferentation (16). The above was a description of activity-based sensory re-education. Selective deafferentation is not recommended because it could affect the sensory input of the healthy side.

Traditional sensory re-education involves reestablishing impaired afferent sensory pathways through interactions with the environment to subsequently restore more discriminative senses. The European Consensus on Sensory Retraining in Peripheral Hand Neuropathy provides a systematic strategy to promote reeducation of tactile cognition by interacting with different textures, temperatures, forms, and objects (17). Although sensory abnormalities following brachial plexus injury are not just present in the hand, it is still feasible to employ the traditional methods of sensory re-education mentioned above. Traditional sensory re-education has mostly been proven to be successful as a participating component in comprehensive rehabilitation treatment, but there are unfortunately no clinical investigations on its effectiveness with sufficient sample numbers.

Mirror visual feedback (MVF) is the process of superimposing the motion picture of the healthy hand on the location of the affected hand using a mirror that has been placed vertically on a table. This creates a motor-sensory connection between the hands (18). Such visual input promotes upper extremity mobility and sensory rehabilitation in children with Duchenne-Erb syndrome, according to a randomized controlled trial in children ages 6–12 years (19). It is possible to treat adults with the same technique. However, a randomized controlled trial with insufficient information on sensory or motor recovery found that MVF combined with TDCS over 3 months may lessen neuropathic pain after brachial plexus injury (20). Chen et al. demonstrated the effectiveness of MVF in sensory and motor reeducation, observing improved learning in hand and finger dexterity compared to traditional sensory. Additionally, patients who used MVF demonstrated increased activation in ipsilateral brain areas and the multimodal association cortex on fMRI (21). Unfortunately, Chen only examined 6 cases, which is not representative, and it is advised that future research include more cases and additional follow-up. MVF is a potential therapy for sensory re-education with brachial plexus injury in adults, although its efficacy has not been explicitly validated and requires additional investigation.

Cross-modal sensory replacement translates tactile information across several modalities using sensing technologies (22). Patients must acquire extra sensor systems, which are not commonly employed in the treatment of brachial plexus injuries. It appears to be a possibility to explore persistent sensory abnormalities with brachial plexus injury. We are delighted to see sensing technologies advance and become more widely used.

#### 3.1.4. Neurotrophic treatment

Once brachial plexus injury has been diagnosed, neurotrophic treatment will be used continually and regularly. B vitamins, mecobalamin (23), citicoline, and other pharmaceuticals that are routinely used in clinical practice are the principal neurotrophic medications. Exogenous neurotrophic factors are also a research hotspot in animal studies due to their extensive effectiveness, low risk of side effects, and high level of safety. It has been demonstrated that topical anti-NGF therapy may reduce chronic neuropathic pain, whereas early topical administration of NGF and CNTF-like medicines may assist in preventing the degeneration of injured nerves (24). Regrettably, there have been fewer exogenous neurotrophic factor clinical medication studies overall, and only enkephalin has received marketing approval. A more comprehensive list of neurotrophic factors with potential neuroprotective effects on motor neurons has been published in research and may be a viable pharmaceutical therapy for brachial plexus nerve injury (25). Furthermore, additional clinical investigations are needed to prove the effectiveness of medications such as gangliosides, hormones, Chinese herbs, etc.

#### 3.1.5. Modalities

Numerous studies have demonstrated the effectiveness of early modalities in reducing swelling, promoting edema absorption, relieving pain, and releasing adhesions. This therapy can be applied in a variety of settings, including ultrashort wave, infrared, and low-energy laser irradiation (15).

Electrical nerve stimulation promotes nerve healing and regeneration and is a useful tool. Depending on the stimulation site, neural electrical stimulation can be classified as deep brain stimulation (DBS), transcutaneous neuromuscular electrical stimulation (NMES), spinal cord stimulation, and transcranial direct current stimulation (tDCS) (26). Neuromuscular electrical stimulation, which is frequently utilized for motor and sensory reeducation in the treatment of children and adults with brachial plexus injuries, has been demonstrated to excite both motor nerve fibers and afferent sensory nerve fibers (27). In contrast, DBS, TDCS, and spinal cord stimulation are more commonly applied to alleviate neuropathic pain. The stimulation techniques mentioned above may need to rely on relatively unharmed nerve-down routes to effectively stimulate peripheral nerves, particularly in individuals with diminished fine hand function following brachial plexus injury. Given the aforementioned, there is a huge market and development potential for an implanted peripheral nerve stimulator that could work directly on nerve branches in the axilla while also providing long-term relief from peripheral neuropathic pain following brachial plexus injury (28).

In addition, transcranial magnetic stimulation (TMS) is often used to collect motor-evoked potentials to assess neurological activity or to treat neuropathic pain as a complement to Noninvasive electrical brain stimulation (29). It is worth mentioning that the strong magnetic field generated by TMS can be employed for peripheral neuromuscular regulation in addition to agitating the central nervous system. Consequently, TMS has the potential to replace transcutaneous neuromuscular electrical stimulation with a technique that has a higher safety profile, and it may also represent the future of neuromagnetic stimulation research.

The benefit of neuro-electromagnetic stimulation in the treatment of neuropathic pain, which may be regularly utilized in pain management, should not be overlooked. However, further clinical investigations and systematic assessment research are required to better understand the suggested paradigms due to the diversity of stimulation paradigms resulting in varying effectiveness.

#### 3.1.6. Traditional Chinese medicine treatment

Due to their capacity to stimulate the repair and regeneration of injured nerves, enhance local blood circulation in the treated area, and lessen postoperative pain, acupuncture and tui na are widely used in traditional Chinese medicine to treat brachial plexus nerve injury. Whereas using tui-na or acupuncture alone is preferable to using medication isolated, the combined rehabilitation impact of both with other treatment modalities is noticeably superior to using tui-na or acupuncture alone. Direct comparisons of various tui na massage or acupuncture point therapies are also scarce. Electroacupuncture was initially shown to be more successful than medication therapy alone in treating 54 individuals with peripheral nerve damage in 1995 (30). A further animal investigation revealed that electroacupuncture therapy may protect against brachial plexus injury by lowering the expression of nNOS and slowing the degeneration of injured neurons (31). A series of case reports that were conducted afterward also supported this finding, making the efficacy more solid. Electroacupuncture can be suggested as a conservative treatment for brachial plexus injury in the absence of pertinent contraindications.

#### 3.1.7. Psychosocial support

Emotional and social factors may influence rehabilitation after brachial plexus injury, particularly in a productive young male population. Their high expectations of efficacy lead to an inability to accept the possibility of permanent disability and the long recovery time required for neurological recovery, making them prone to psychological disengagement, denial, and venting behaviors in the later stages of treatment, and highly cooperative in the earlier stages. As a consequence, it is essential to regularly monitor patients’ adherence to treatment during the later phases of recovery, prevent dangerous behaviors, and promptly offer psychosocial assistance such as psychotherapy, psychiatric medication, and family support. For the time being, there is no systematic psychological rehabilitation strategy for brachial plexus injury, and few studies on psychological rehabilitation have been conducted. Studies on psychological aspects in brachial plexus injury patients have demonstrated that these individuals typically employ adaptive coping strategies and self-distraction in the early phases of their recovery to deal with negative psychology (32). Therefore, we investigated pertinent psychological rehabilitation models and came to the conclusion that active positive mindfulness therapy, such as meditation, body scanning, and other forms of active positive mindfulness, can support the above-mentioned positive psychological strategies and lead to a positive psychological state, which reduces stress and eradicates depression. Future research on the effects of various social support measures on the functional rehabilitation and psychological condition of brachial plexus injury patients is advised.

### 3.2. Rehabilitation in the acute postoperative period

Before starting a structured rehabilitation program after surgery, the muscles and nerves must have had enough time to recover. Controlling pain and edema in the acute postoperative period is the main therapy objective (9). Postoperative patients with complaints of pain might involed pain management because quick pain relief can enhance the patient’s psychological circumstance and speed up recovery. Potential pain relief options include opioids, topical analgesic patches, antiepileptic drugs, antidepressants, and nerve electrical stimulation, according to a systematic review of pain management for complicated nerve injuries (4). There is limited graded pain evaluation after brachial plexus injury, resulting in a wide range of pain management therapy options. Procedures for recommended pain rehabilitation management are not arranged in a hierarchical order. It is critical to highlight that pain control must be utilized throughout the brachial plexus injury treatment procedure.

Additionally, there is general agreement to start early passive mobility of the proximal and distal joints of the trauma and to locally immobilize the nerve anastomosis site in the acute postoperative phase. The purpose is to safeguard the surgical site, promote nerve gliding, and eventually reduce pain and encourage functional recovery. The best period for postoperative fixation is not universally agreed upon; however, it is crucial to hold off until the nerve anastomosis site is tension-free, particularly after repairing a nerve with significant local tension. Based on randomized controlled research of comprehensive postoperative therapy in patients with brachial plexus injuries, the passive motion of the joint within an unrestricted range of motion is advised and needs to be combined flexibly depending on the type of surgery. In conclusion, it is critical to consider the functional recovery of the primary injury site as well as the remaining neuromuscular functional compensation after a surgical transfer, such as after a phrenic nerve transfer to the anterior branch of the superior brachial plexus, which requires both flexions of the elbow joint and deep inspiration (33). Numerous randomized controlled studies have advised thorough rehabilitation following surgery using therapeutic modalities that do not significantly diverge from those performed in spontaneous recovery (34).

### 3.3. Rehabilitation in the sequela period

Even with complete rehabilitation and satisfactory neurological recovery, associated complications, including pain, muscle atrophy, restricted joint mobility, and subsequent abnormalities, are still possible (15). Considering that complete alleviation of brachial plexus injury is challenging with rehabilitation alone in the sequela period, surgical procedures such as neuroma removal to reduce pain and latissimus dorsi tendon grafting to the rotator cuff to address shoulder mobility limitations are typically employed (35). Postoperative rehabilitation strategies for sequelae are not contradictory and may be adapted to acute postoperative rehabilitation for brachial plexus injuries. The distinction is that patients need to be reevaluated for postoperative immobilization duration and joint range of motion since they are generally related to a loss in muscle performance and joint function. Regrettably, there is no clinical consensus on these specifics (36). Treatment methods may rely on the expertise of experienced physicians and therapists. Power-assisted orthoses can offer a functional replacement in the end phase and have some effectiveness in motor re-education when the surgical benefit is not preferably factored in (37). Rehabilitation of brachial plexus injuries in the sequela period seems to be a neglected area that needs further development.

## 4. Neonatal brachial plexus injury rehabilitation

Clinical rehabilitation and research on brachial plexus injuries in children have been widely conducted, particularly in children aged 1–12 years. However, the rehabilitation treatment of neonatal brachial plexus injury (NBPI) confronts challenges such as poor patient compliance, difficult efficacy assessment, and high family participation, resulting in slow advancement and becoming an insurmountable barrier for brachial plexus injury. It’s the fact that some demographics indicate a gradually declining frequency of brachial plexus injury following a neonatal birth injury, because of fewer forceps used (38). First, neonatal brachial plexus injuries can cause long-term chronic damage and necessitate early intervention for rehabilitation; second, a high level of home rehabilitation demands specific rehabilitation instructions for parents in preparation. The basic stage in diagnosing NBPP is clinical evaluation and physical examination by an expert doctor. Next, fewer intrusive procedures, frequently magnetic resonance imaging (MRI) and ultrasound scanning (US), are needed. If the diagnosis is still elusive, electrodiagnosis can be utilized (39). The anatomy of a neonate (0–28 days) is less developed than that of an adult; thus, precise localization is more crucial than in adults. Knowing the location and severity of a nerve injury can also help to accurately guide reconstruction procedures and provide advice to families.

For neonates with a clear diagnosis, early referral to an integrated multidisciplinary management model is advised by several systematic reviews of NBPP rehabilitation. The fundamental rehabilitation strategies advised are parallel to those for adults, which include treatments such as passive/active motor exercises, smooth joint motor, sensory stimulation, electrical stimulation, and botulinum toxin injections (40). To serve as a reference, we evaluated the available literature and collected the therapy recommendations that, when applied to newborn brachial plexus injury rehabilitation: firstly, cooperation between the family and the newborn should be considered when selecting a rehabilitation process. As the family rehabilitation model predominates and is more suitable for the care of the infant, simple, straightforward, and repeatable treatments can be selected to allow parents to perform rehabilitation therapy numerous times during the day; Secondly, rehabilitation should intentionally select to encourage active movement to restore function to the injured limb rather than compensating for the healthy side by emphasizing developmental neglect and misuse related to neonatal growth and development (41). In conclusion, there is variability in the efficacy of different treatment methods, which calls for more study to clarify. The agreement on a comprehensive rehabilitation process for NBPP continues to be in the early stages of development.

## 5. Discussion

**Figure 2:**
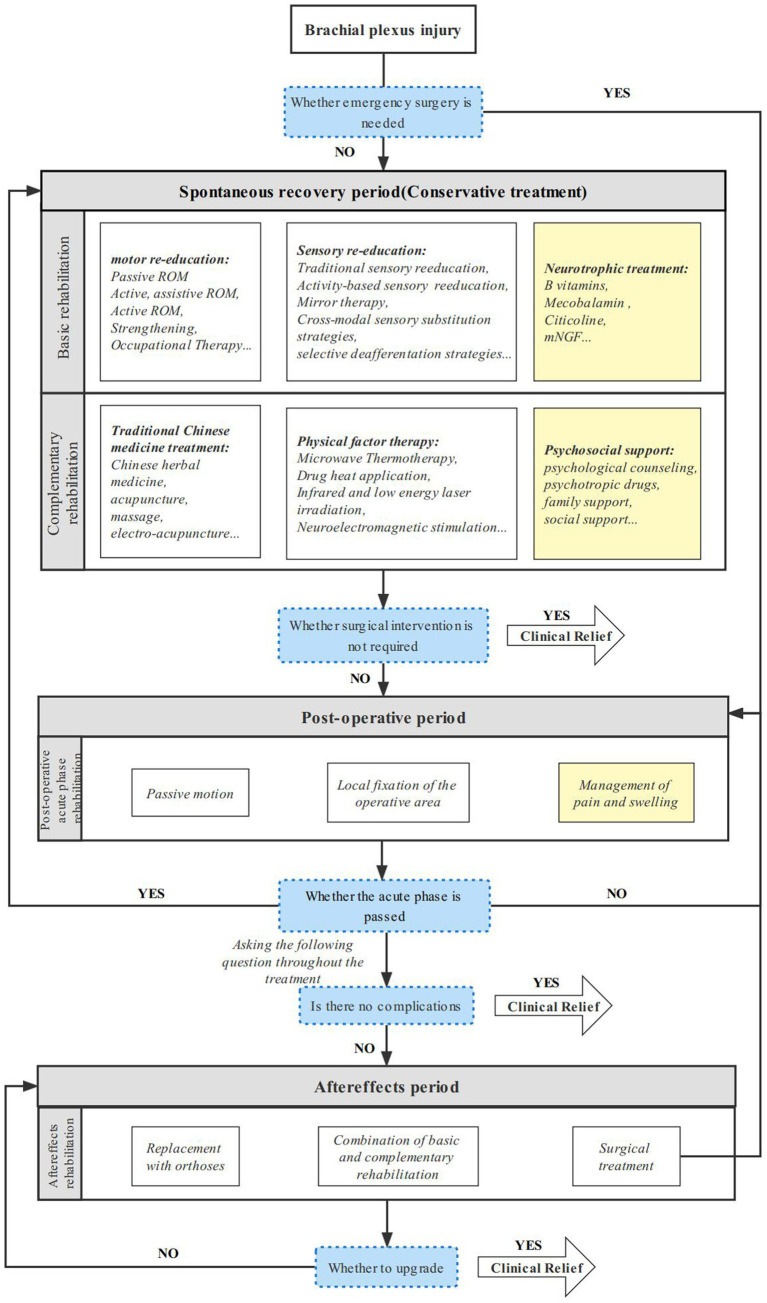
Comprehensive rehabilitation protocol for brachial plexus injury. A sustainable and comprehensive rehabilitation protocol for adult brachial plexus injury in different periods.

The functional recovery of brachial plexus injury is a worldwide challenge. This study compiled the various rehabilitation modalities for clinicians, arranged them into rehabilitation protocols from the time a brachial plexus patient was seen until clinical remission (Figure 2) and enabled the systematic management of BPI patients. A fairly comprehensive evaluation of physical therapy for brachial plexus injuries has been conducted (42). On this basis, we have closely followed the latest advances in current clinical research on brachial plexus injury rehabilitation, and we summarize its intervention strategies and outcomes, details see Table 1.

**Table 1:** Characteristics of the clinical studies on rehabilitation of brachial plexus injuries published in recent years.

Author/year	Study location	Study design/population	Level/time of injury	Intervention	Main outcomes
(46)	Institute for Nursing and Interprofessional Research, Children’s Hospital Los Angeles, Los Angeles, CA	Randomized crossover trial *n* = 21 (11 males, 10 females) Age = 25 ± 10.3 mo	Unilateral NBPP uninformed	Group CIMT: 3 weeks of casting the unaffected limb followed by 5 weeks of transference activities. Group standard: usual occupational therapy for 8 weeks	Assisting Hand Assessment (AHA) The Pediatric Motor Activity Log-Revised (PMAL-R) Group CIMT is favored over group standard care for bimanual activity performance.
(47)	University Institute of Physical Therapy, University of Lahore, Lahore, Pakistan	Prospective randomized clinical trial *n* = 66 Unrealized	Uninformed <3-month	Group 1: ultrasound therapy 2 days a week for 6 weeks(using a pulsed mode 0.8 W/cm^2^ and frequency 1 MHz), wrist splinting, tendon gliding exercises Group 2: ultrasound, splinting, tendon gliding exercises, a neuromobilization technique.	ROM, manual muscle strength, pain at NPRS, functional status on the Boston Carpal Tunnel Syndrome Questionnaire (BCTQ) -symptom severity scale (SSS), functional status scale (FSS) Group 2 showed a statistically more significant increase in flexion, extension, decrease in pain, decrease in SSS, decrease in FSS, and BCTQ as compared to the group 1.
(21)	School and Graduate Institute of Physical Therapy, College of Medicine, National Taiwan University, Taipei, Taiwan	Prospective randomized clinical trial *n* = 6 Uninformed	Median and/or ulnar nerve injury Uninformed	Group 1: mirror-therapy (using mirror reflection of the unaffected hand in order to train the affected hand) Group 2: sensory-relearning	Clinical: Semmes Weinstein Monofilaments (SWM) test, static 2-point discrimination test (S-2PD), grip strength, the Disabilities of the Arm, Shoulder and Hand (DASH) scores Radiographic: fMRI Group 1 had better outcomes in finger dexterity and manual dexterity, and fMRIs showed greater activation in the multimodal association cortices and ipsilateral brain areas during motor tasks.
(48)	School of Physiotherapy, King Edward Medical University, Lahore	Prospective double blinded randomized control trial *n* = 66 Age = 37.79 ± 5.91 years	Carpal tunnel syndrome <3-month	Group 1: ultrasound therapy, wrist splinting, tendon-gliding exercises Group 2: ultrasound therapy, wrist splinting, tendon-gliding exercises, neuromobilisation. Wrist splinting: the wrist was repeatedly moved into and out of stretch by performing a few degrees of flexion and extension at the wrist.	Boston Carpal Tunnel Syndrome Questionnaire (BCTSQ)--symptom severity scale (SSS), functional status scale (FSS) Group 2 showed better index of BCTSQ compared to Group 1
(45)	Department of Rehabilitation, Leiden University Medical Center, Leiden, Netherlands	Randomized controlled trial *n* = 55 Unrealized	Elbow flexion contracture of ≥30° uninformed	Group 1: night-worn dynamic orthosis for 1 year Group 2: serial casting for 1 year	Elbow flexion contracture Goal attainment scaling Both treatment options showed a comparable reduction in the elbow flexion
(49)	Mexican Faculty of Medicine of La Salle University, Mexico City, Mexico	Prospective, longitudinal, nonrandomized, self-controlled before and after study *n* = 10 Age = 34 ± 16.85 years	BPI of traumatic etiology, high level of injury (proximal third of the upper extremity), and refractory neuropathic pain (medical treatment with at least 2 different analgesic drugs during 3 months of management) Uninformed	Surgical neurolysis	Visual Analog Scale (VAS) British Medical Research Council (BMRC) scale 9 patients showed improvement in the continuous and paroxysmal pattern of pain.
(6)	Department of Anesthesiology and Pain Medicine, Chungnam National University Hospital, Daejeon, Korea	Prospective non-randomized controlled trial *n* = 134 Uninformed	Uninformed	Group 1: Continuous interscalene brachial plexus block (CISB)after arthroscopic surgery for rotator cuff repair Group 2: single interscalene brachial plexus block(SISB) combined with intravenous patient-controlled analgesia (IV-PCA) after arthroscopic surgery for rotator cuff repair	Quality of recovery (QoR-40) score CISB showed a higher quality of recovery score than SISB with IV-PCA in arthroscopic rotator cuff repair
(50)	The Division of Anesthesia, Intensive Care, and Pain Management, Tel Aviv Medical Center, Sackler Faculty of Medicine, Tel-Aviv University, Tel Aviv, Israel	Randomized Clinical Trial *n* = 59 Uninformed	Uninformed	Group 1: perioperative oral pregabalin (Lyrica, twice daily starting the evening before surgery, for a total of 4 doses) Group 2: single-shot ISBPB (20 mL of bupivacaine 0.25%)	Median self-reported pain score (on a visual analog scale of 0 to 100) pain during activity, postoperative opioid consumption, opioid-related adverse effects, quality of recovery, and pain satisfaction score Perioperative use of pregabalin in adults undergoing arthroscopic RC repair provided analgesia comparable to that of ISBPB for 10 days after surgery.
(51)	Physical Medicine and Rehabilitation, Health Science University Antalya Training and Research Hospital, Antalya, Turkey	Randomized, prospective clinical study *n* = 143 Uninformed	Clinical features (such as pain and/or numbness in the median nerve distribution or nocturnal pain) >3-month	Group 1: low level laser therapy (LLLT) Group 2: corticosteroid injection	Numbness and pain, QuickDASH questionnaire, grasping tests, Tinel and Phalen tests electrophysiological tests: Nerve conduction studies (NCS) Radiographic: MRI
(52)	Centre for Interdisciplinary Research in Rehabilitation of Greater Montreal, Institut universitaire sur la réadaptation en déficience physique de Montréal, Centre intégré universitaire	Randomized controlled study *n* = 30 Age = 54.2 ± 9.2 years	Diagnostic of CTS *via* an electrodiagnostic test Uninformed	Group 1: four-week home-based neuromobilization exercise group(NEP) Group 2: standard care	Feasibility: recruitment, attrition, adherence, satisfaction, and safety efficacy metrics: median nerve integrity and neurodynamics, tip pinch grip, pain, and upper limb functional abilities novel preoperative neuromobilization exercise program has limited beneficial effect.
(53)	Department of Physical Medicine and Rehabilitation, Tri-Service General Hospital, School of Medicine, National Defense Medical Center, Neihu, Taipei, Taiwan, Republic of China	Randomized Double-Blind Clinical Trial *n* = 35 Unrealized	Mild or moderate CTS >3-month	Group 1: one ultrasound-guided perineural injection of 2.5 mL hyaluronic acid (HA) Group 2: 2.5 mL normal saline injection through in-plane, long-axis approach to separate the median nerve from the flexor retinaculum *via* nerve hydrodissection	Boston Carpal Tunnel Syndrome Questionnaire (BCTQ) scores, e numeric rating scale (NRS), electrophysiological domains, and the cross sectional area of the median nerve A single ultrasound guided perineural HA injection may have short-term therapeutic efficacy for mild or moderate CTS.
(54)	Department of Physiotherapy, Babol University of Medical Sciences, Babol, Iran	Randomized clinical trial *n* = 60 Unrealized	Mild or moderate CTS Uninformed	Group 1: 1500 shocks on the carpal tunnel and conventional physiotherapy for 10 sessions Group 2: 1500 shocks on the carpal tunnel and median nerve pathways and conventional physiotherapy for 10 sessions Group 3: conventional physiotherapy for 10 sessions	Pain and paresthesia intensity sensory and motor distal latency Radial shockwave combined with conventional physiotherapy is an effective noninvasive treatment for mild-to-moderate carpal tunnel syndrome.
(55)	Department of Physical Medicine and Rehabilitation, Tri-Service General Hospital, School of Medicine, National Defense Medical Center, No. 325, Sec. 2, Cheng-Kung Road, Neihu District, Taipei, Taiwan, Republic of China.	Prospective randomized, single-blind trial *n* = 47 Unrealized	Mild or moderate CTS Uninformed	Group 1: short-axis hydrodissection Group 2: long-axis hydrodissection	Boston Carpal Tunnel Syndrome Questionnaire (BCTQ) score, the cross-sectional area of the median nerve and electrophysiological studies. Both short-and long-axis hydrodissection are effective for patients with mild-to moderate CTS
(56)	Department of Rehabilitation, Radboud university medical center, Donders Institute for Brain, Cognition and Behavior, Nijmegen, the Netherlands	Randomized Controlled Trial *n* = 27 Unrealized	Neuralgic amyotrophy (NA) caused by auto-immune inflammation of nerves in the brachial plexus territory Uninformed	Group 1: multidisciplinary rehabilitation Group 2: usual care multidisciplinary rehabilitation: a 16-week outpatient multidisciplinary rehabilitation program involved 1 h of physical therapy and 1 h of occupational therapy per day. 4-weekly treatment sessions followed by two sessions every other week and two monthly sessions.	Clinical: a hand laterality judgment task Radiographic: task-based functional MRI.

Currently, a significant proportion of patients with brachial plexus injury are young adults and newborns, and these patients usually have an expectation of immediate recovery. However, brachial plexus injury typically requires a lengthy recovery period due to the complexity of the injury and the slow recovery tempo. During long-term rehabilitation, the degree of neurological recovery (including motor and sensory recovery, etc.), psychological status, and complications of the affected limb vary widely, requiring the clinician to develop an individualized rehabilitation program, which requires a great deal of experience. Therefore, patients may be in various stages of rehabilitation at the time of consultation, and there is no clinical consensus on how to manage patients in each stage in an integrated manner, making it easy to overlook the various aspects of rehabilitation principles that require attention at various stages of disease development. We will do some sorting later to fill in the gaps in this section.

The efficacy of comprehensive rehabilitation therapy is well established. For patients with brachial plexus who do not need emergency surgery and proceed to the spontaneous recovery stage of rehabilitation. Local external fixation support, motor and sensory re-education, and neurotrophic therapy are common alternatives. When specific treatment requirements are met, physical factor therapy, traditional Chinese medicine treatment, psychosocial support, and other supplementary treatment methods can be applied. For patients undergoing postoperative acute rehabilitation postemergency surgery, passive movement of the afflicted limb and nerve removal site, with suggested mobility within the unrestricted joint range, can be used to reduce pain and edema. For further rehabilitation, conservative therapy options available throughout the spontaneous recovery period may be selected. It is impossible to totally eliminate complications, and when they do arise, the patient moves into the sequelae phase of rehabilitation. These complications include muscular atrophy, joint stiffness, chronic neuropathic pain, etc. To accomplish functional compensation and improve the patient’s quality of life, an end-stage rehabilitation approach might be adopted, mostly employing vigorous replacement therapy. Neurotrophic therapy, pain and swelling control, and psychosocial support can all be consistently employed at different phases because pain and psychiatric illnesses might flare up at various periods. Complete recovery from brachial plexus injury is exceptional, but when clinical remission—that is, increased performance and quality of life—is attained, the patient feels sufficiently better to jump their treatment protocol and return to home rehabilitation. This page also provides an overview of the fundamental principles of rehabilitation for generally slow-progressing neonatal brachial plexus injuries, which can help to hasten the development of treatment approaches to this specific demographic problem.

Artificial intelligence and rehabilitation techniques have been developed in the last 3 years. An electroencephalography (EEG)-based human-machine interface combined with contralateral C7 transfer surgery can treat brachial plexus injury, but further research is required to understand how it promotes cortical remodeling (43). Virtual reality and robotics combined to facilitate upper extremity pain management and sensory recovery after brachial plexus injury (44). The very first mention of dynamic orthoses as having a role in motor re-education came in 2018 (37), but more recent research has revealed that dynamic nocturnal orthoses are equally effective as continuous cast immobilization in treating elbow flexion contractures in kids with brachial plexus nerve birth injuries (45). As children’s upper extremities move less than those of adults, dynamic orthoses may only serve as a fixation device in children with brachial plexus injuries while offering more motor support in adults. Individual rehabilitation is a constant topic, and AI rehabilitation has a higher degree of adjustability, sure AI involvement will become an unstoppable trend.

The literature review deserves some essential conclusions and insights to be presented. The lack of sufficient case studies, inadequate follow-up time, the lack of a recognized control group, and the propensity to employ comprehensive rehabilitation as an intervention to improve treatment results are more or fewer defects of the literature on brachial plexus injury treatment methods. Therefore, techniques like stem cell therapy that remain at the level of animal trials are not included in the clinical approach presented in this work. Our work excludes the mention of efficient treatment methods in case reports involving a small number of patients but keeps useful comments. This takes into account treatment approaches that have all been shown in numerous case reports to be successful. Randomized controlled studies reporting effective rehabilitation treatment modalities and clinical studies with a long-term follow-up process were considered first. The rehabilitation protocols described in this article are experimental and can be supplemented with additional clinical treatment modalities that show benefits. Unfortunately, due to a lack of quantitative assessment methods, this article only develops a comprehensive treatment protocol rather than offering a suitable rehabilitation therapy approach for individuals with varying injury severity and locations. The determination of the optimal treatment approach for a specific patient is still left to the discretion of qualified medical staff and therapists. In summary, it is strongly advised that future research focus on the efficiency of potential comprehensive rehabilitation programs in the management of BPI.

## 6. Conclusion

The efficacy of comprehensive rehabilitation therapy is well established. Physical therapy, traditional Chinese medicine treatment, psychosocial support, and other supplementary treatment methods can be applied when someone undergoes a brachial plexus injury, in addition, artificial intelligence and rehabilitation techniques are popular in these years which need further research. All the rehabilitation protocols we described are experimental and they show benefits as supplemented with additional clinical treatment modalities. In summary, we strongly advised that future research focus on the efficiency of potential comprehensive rehabilitation programs in the management of BPI, which will facilitate the return of patients, physical activity levels.

## Author contributions

HL: visualization and methodology. JC: writing—review and editing, conceptualization. JW: writing—original draft, visualization. TZ: supervision. ZC: authors may have contributed in multiple roles. All authors contributed to the article and approved the submitted version.

## Conflict of interest

The authors declare that the research was conducted in the absence of any commercial or financial relationships that could be construed as a potential conflict of interest.

## Publisher’s note

All claims expressed in this article are solely those of the authors and do not necessarily represent those of their affiliated organizations, or those of the publisher, the editors and the reviewers. Any product that may be evaluated in this article, or claim that may be made by its manufacturer, is not guaranteed or endorsed by the publisher.
